# Interdisciplinary Treatment of a Case With Unilateral Cleft Lip and Palate in the Mixed Dentition

**DOI:** 10.7759/cureus.37148

**Published:** 2023-04-05

**Authors:** Abhishek D Sanchla, Sunita Shrivastav, Nitin D Bhola, Ranjit Kamble

**Affiliations:** 1 Department of Orthodontics, Sharad Pawar Dental College & Hospital (SPDCH), Wardha, IND; 2 Department of Oral and Maxillofacial Surgery, Sharad Pawar Dental College & Hospital (SPDCH), Wardha, IND

**Keywords:** interceptive orthodontics, cleft orthodontics, alveolar bone grafting, maxillary expansion, cleft lip and palate

## Abstract

A 10-year-old girl had reported to Sharad Pawar Dental College. Her parents had chief complaints of lip and palate deformity. On examination, it was found that the patient had unilateral cleft lip and palate on the right side. The aim was to expand the maxilla with alveolar bone grafting in the cleft region to facilitate the eruption of permanent canine and further reduce the deformity to prepare the patient for face mask therapy, reduce morbidity in the permanent dentition, and avoid Le Fort one surgery in the future. She had been previously operated on for cleft lip repair and palatal fistula closure eight years back. The present condition in the mixed dentition needed arch expansion, bone in the cleft region for the eruption of permanent canine, and further arch alignment for facemask therapy. This would reduce the severity of skeletal deformity and later on avoid the surgical advancement of the maxilla.

## Introduction

A cleft lip is defined as a congenital deformity that occurs in the primary palate, which is located anteriorly to the incisive foramen, and its occurrence may be unilateral, bilateral, complete, or incomplete [1]. The overall pooled birth prevalence (random effect) of orofacial clefts is 4.5 per 1,000 total births, and the incidence is 1.3 per 1,000 births [2]. Transverse expansion of the collapsed maxilla is considered the initial step for management in the mixed dentition to correct the posterior crossbite and accommodate the permanent canine, which would facilitate attaining the desired inter-canine width and proper arch alignment for facemask therapy [3]. Inadequate bone in the cleft region is the prime reason for the inability of permanent canines to erupt [4]. Bone successfully grafted to the alveolar process brings many advantages as it provides continuity and stability of the dental arch, and in cases with missing permanent teeth in the cleft area, prosthodontic work is facilitated. Other advantages include the ability for the eruption of canines and lateral incisors otherwise lost in the cleft [5]. The presence of bone makes it possible to move adjacent teeth orthodontically into the former cleft area [6]. The case presented here describes the treatment protocol followed for a case with unilateral cleft of lip and palate which constituted maxillary expansion followed by secondary alveolar bone grafting and fixed orthodontic therapy. Written informed consent was obtained from the patient's parents before the treatment, and the departmental ethical committee granted all the approvals for the case to be treated and presented.

## Case presentation

**Figure 1:**
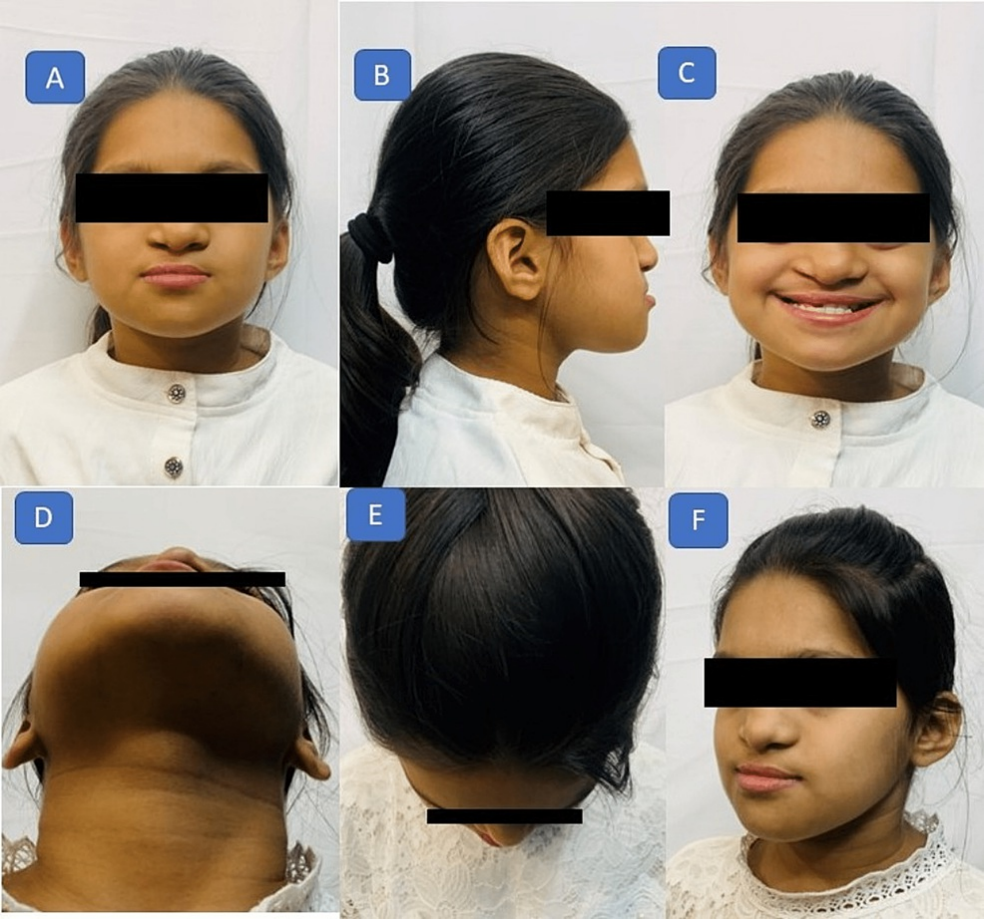
Pretreatment photographs (extraoral): (A) frontal view showing a square facial morphology; (B) profile view showing a convex profile; (C) smiling view showing lower incisor display; (D) worm's view showing diminished zygomatic prominences; and (E) bird's eye view showing diminished malar prominences.

A 10-year-old girl was reported to the department with the chief complaint of poor aesthetics due to cleft lip and palate. She had a history of primary lip repair at the age of one year followed by palatal fistula closure at the age of four. No other relevant medical history was reported. On extraoral examination, a concave facial profile was noticed with a hypoplastic maxilla, diminished malar prominence, and a reduced lower anterior facial height (Figure 1).

**Figure 2:**
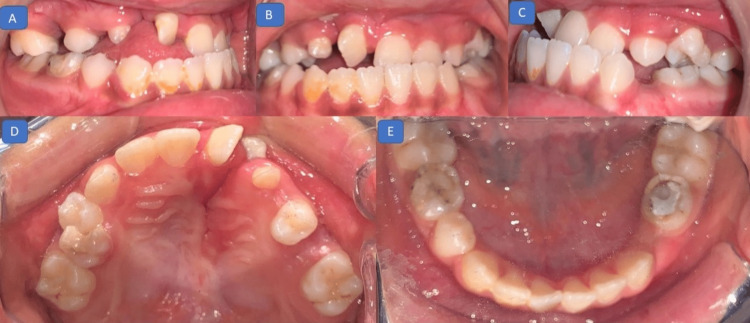
Pretreatment photographs (intraoral): (A) right lateral view showing a class III molar relation; (B) frontal view showing a negative overjet; (C) right lateral view showing a class III molar relation and missing deciduous first molar; (D) maxillary occlusal view showing a rotated right central incisor and discontinuity of alveolar ridge on the right side; (E) mandibular occlusal view showing a well-aligned arch.

On intraoral examination, a class III molar relation with an anterior crossbite of 4 mm and posterior cusp-to-cusp relation were present. The upper arch had a supernumerary tooth in the cleft region in addition to a deciduous canine. The upper incisors were rotated, and the mandibular arch was well aligned (Figure 2).

**Figure 3:**
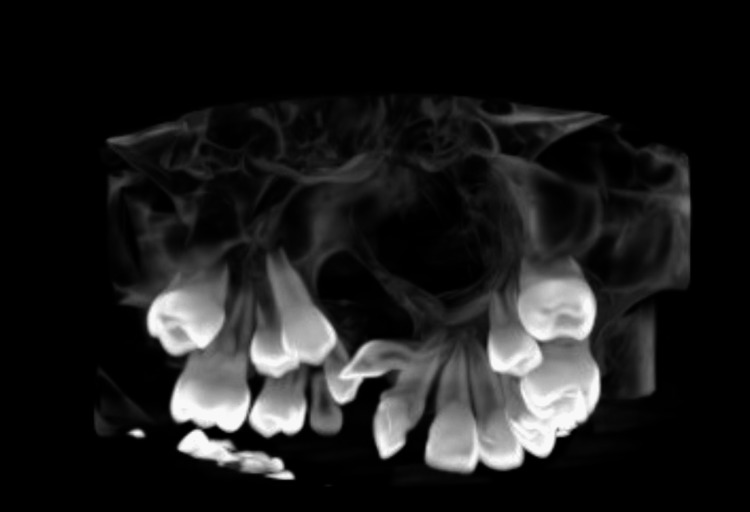
Cone beam computed tomography systems view of the cleft region showing discontinuity of the alveolar bone and the presence of two supernumerary teeth.

Radiographically, on a lateral cephalogram, it was inferred that the skeletal pattern was class III with a horizontal growth pattern. The maxilla was retrognathic with a normal mandible. On the cone beam computed tomography scan, a discontinuity of the alveolar process on the right maxillary segment was observed and two supernumerary teeth were present in the cleft region in addition to a deciduous canine on the right side. A developing permeant canine with an open apex was seen above the cleft region. Erupting mandibular second premolars were also evident (Figure 3).

**Figure 4:**
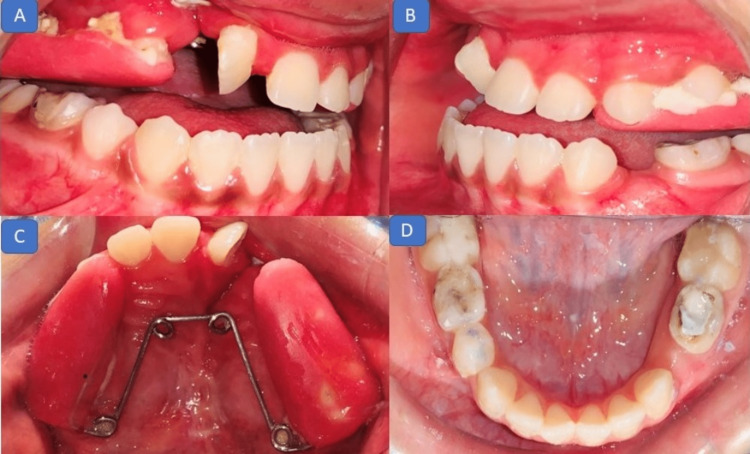
Intraoral photographs (expansion appliance): (A) right lateral view and (B) left lateral view showing a full acrylic coverage expansion plate; (C) occlusal view showing a quad helix expansion plate for bilateral expansion.

The treatment objectives were to correct the posterior crossbite, augment the deficient alveolar bone, and facilitate the eruption of the permanent canine on the right side through the cleft region. The treatment plan included anterior and posterior expansions of the maxilla, extraction of supernumerary teeth, and the deciduous canine in the cleft region, followed by alveolar bone grafting and leveling and alignment of arches after the bone grafting procedure. The treatment progress included the fabrication of a quad helix expansion plate with full coverage of the maxillary posterior dentition to aid in the skeletal expansion (Figure 4).

**Figure 5:**
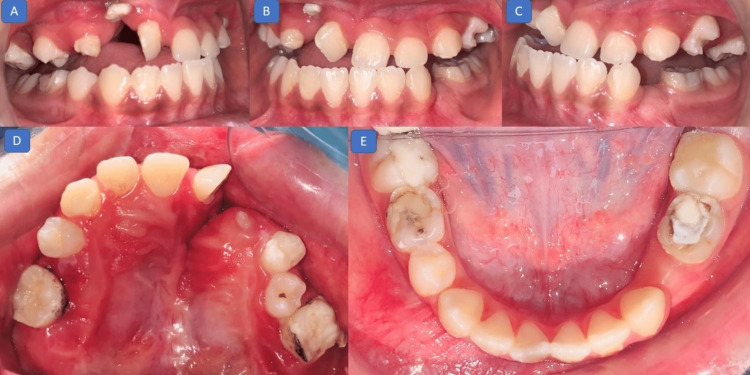
Postexpansion photographs (intraoral): (A) right lateral view showing the correction of the buccal crossbite; (B) frontal view showing the expanded maxillary arch; (C) left lateral showing the correction of the buccal crossbite; (D) maxillary occlusal showing the bilateral maxillary expansion; and (E) mandibular occlusal view showing a stable arch.

The expansion was continued for four months with disarticulation of the bite. Six millimeters of posterior expansion was achieved after four months (Figure 5). Simultaneously, two supernumerary teeth in the cleft region were extracted in the Department of Pediatric and Preventive Dentistry. The case was then referred to the Department of Oral and Maxillofacial Surgery for alveolar bone grafting.

**Figure 6:**
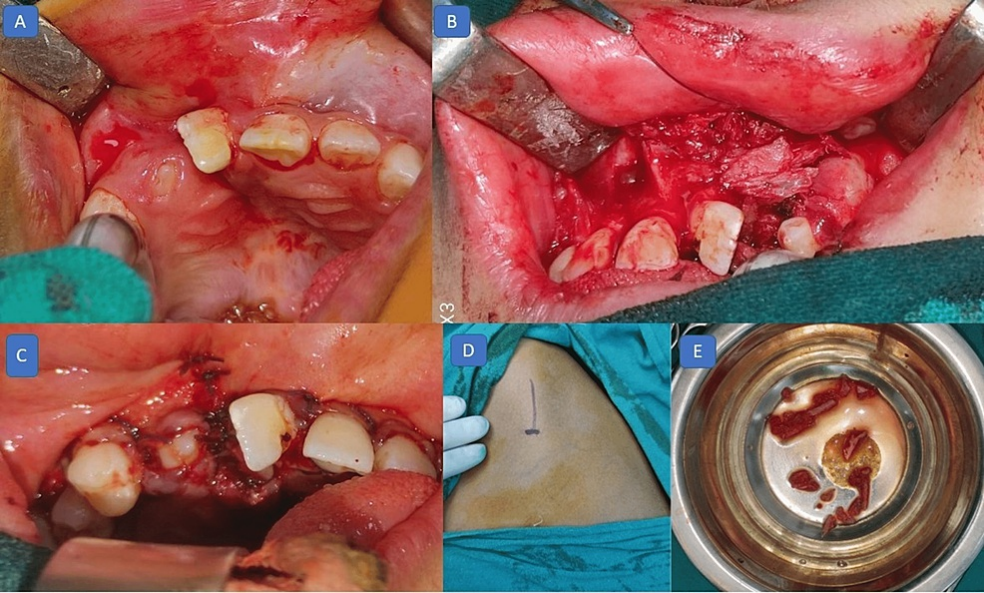
Surgical photographs (alveolar bone grafting): (A) healed surgical area after extraction of supernumerary teeth; (B) surgical exposure of cleft defect; (C) sutured cleft area after alveolar bone grafting; (D) iliac crest incision plan; and (E) donor site graft material.

The left side iliac crest was chosen as the donor site for the graft. The surgical process was as follows: surgical exposure of the cleft area and measurement of the graft site followed by retrieval of graft material from the iliac crest. The graft material was then placed in the cleft region followed by surgical closure with a full-thickness flap (Figure 6). One month of the postsurgical resting phase was provided after surgery. An intraoral occlusal radiograph was taken to check for the take-up of the graft, and the graft region was observed to be healthy, with the absence of bone resorption.

**Figure 7:**
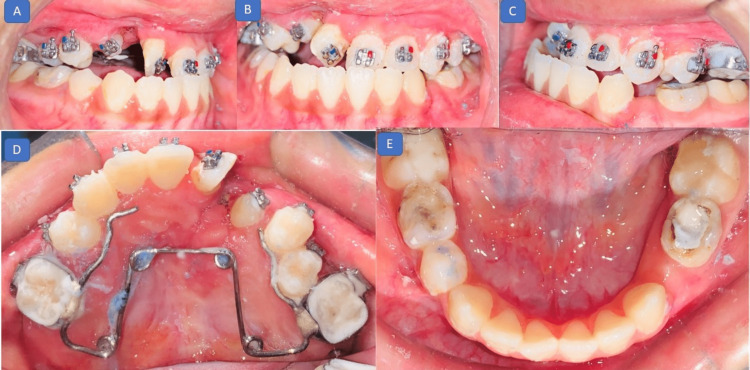
Postsurgical photographs (a fixed appliance with a retention plate): (A) right lateral view showing continuity of alveolar ridge in the cleft region; (B) frontal view showing a healthy tissue for alignment; (C) left lateral view; (D) maxillary occlusal view showing passive quad helix expansion appliance for retention; and (E) mandibular occlusal view showing a well-aligned and stable arch.

A passive quad helix expansion plate was given as a retention plate to maintain the expansion. Four months later, this was followed by upper and lower arch bonding (Figure 7). Initially, alignment was started with 0.014-inch nickel-titanium wires in both the arches followed by 0.016- and 0.018-inch nickel-titanium wires. 

**Figure 8:**
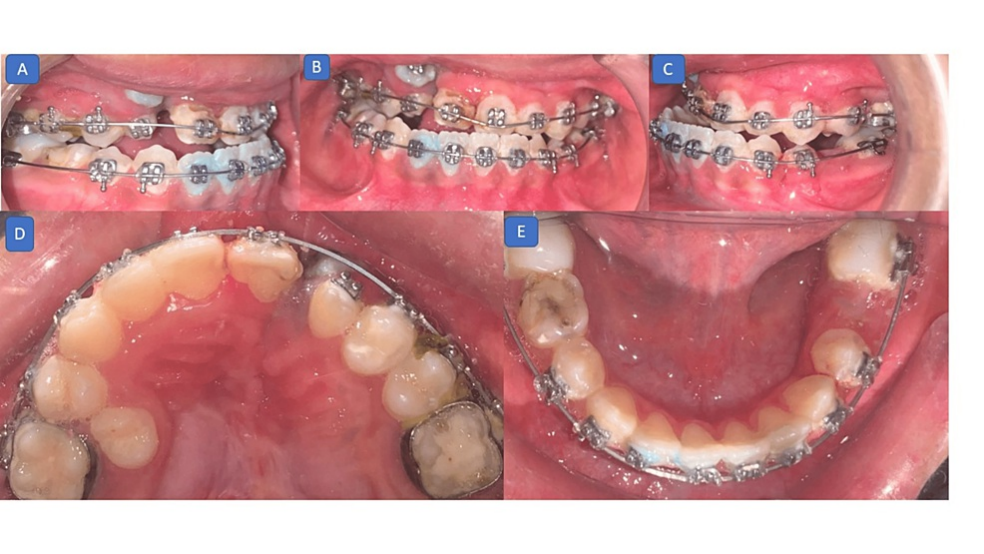
Current photographs (intraoral): (A) right lateral view showing erupting permanent canine through the cleft region; (B) frontal view showing well-aligned maxillary incisors; (C) right lateral view showing a class III molar relation and missing deciduous first molar in the mandibular arch; (D) maxillary occlusal view showing a well-aligned arch with continuity of alveolar ridge and erupting permanent canine on the right side through the bone-grafted region; (E) mandibular occlusal view showing a well-aligned arch.

Currently, the case is on 17 inch × 25 inch nickel-titanium wires in both arches. The alignment of the upper and lower arches has been improved with the continuity of the alveolar bone. The permanent canine on the right side was partially erupted citing the maxillary expansion's success (Figure 8).

Further maxillary protraction with a Delaire facemask is planned with the continuation of leveling, alignment, and space closure. This early intervention was planned to minimize the need for orthognathic surgery in the future.

## Discussion

Although popular, expansion and iliac crest bone grafts did not affect the overall treatment results in unilateral cleft lip and palate cases [7]. However, over the years, the evidence obtained through various clinical studies has pointed to better treatment results in cases intercepted early during their mixed dentition by expansion followed by alveolar bone grafting. This was supported by researchers who reported that secondary bone grafting after expansion of the maxilla was the most popular practice among oral surgeons and others who reported that the iliac crest was considered to be the best donor site for an alveolar bone graft. It helped in the stabilization of the cleft maxilla after expansion and aided in maintaining the continuity of the arch [8,9]. Clinicians radiographically proved similar advantages of secondary bone grafting with expertise in cleft care [10]. Maxillary arch expansion is needed to loosen up the circum-maxillary sutures to further protract the maxilla in further treatment. Our case was intercepted at 10 years of age, and similar treatment timings between 8 and 12 years were advised by authors who extensively studied treatment timings in cleft cases [11,12]. Before the alveolar bone grafting, a quad helix expansion plate was used in this case. Bell and LeCompte recommended using a quad helix expansion appliance for expansion in cleft lip and palate cases; they also reported an average increase of 3.62 and 6.70 mm in the maxillary cuspid and molar region, respectively, with its use. They also explained the need for pre-grafting expansion in cleft cases for the accomplishment of skeletal segmental movement at the expense of expanding the cleft area [13]. Weissler et al. advised assessment of graft take-up before initiation of any orthodontic therapy and a waiting period of four to six months before initiation of orthodontic alignment [14]. A similar protocol was followed in our case wherein a full acrylic coverage quad helix appliance was used for bilateral skeletal maxillary expansion. This aided in the correction of posterior crossbite and loosened up the circum-maxillary sutures for further maxillary protraction. Simultaneously, the supernumerary teeth in the cleft region were extracted and sufficient time was provided for wound healing before alveolar bone grafting, which resulted in healthy graft take-up. Leveling and alignment phase witnessed the spontaneous eruption of the permanent canine through the grafted bone in the cleft region. The partially erupted canine on the right side would be further aligned by the piggyback technique. All the early measures undertaken would improve the prognosis of facemask therapy. Cases with cleft lip and palate require a multidisciplinary management protocol with as early intervention as possible. The aim during each phase of treatment should be directed toward reducing morbidity and improving the quality of life of patients. The results of our case demonstrated that when all the recommended procedures are followed by orthodontic treatment, morbidity in cleft cases can be minimized while improving their treatment prognosis.

## Conclusions

Treatment of cleft lip and palate requires a comprehensive treatment protocol to effectively treat cases at various stages of development and reduce morbidity while aiming for the best aesthetic and functional results. This includes expansion of the collapsed maxillary segment followed by alveolar bone grafting and fixed orthodontic therapy for leveling and alignment. As a result, maxillary protraction becomes feasible, and the chances of a need for surgical maxillary expansion are minimized.
